# Blood cadmium levels and Alzheimer’s disease mortality risk in older US adults

**DOI:** 10.1186/s12940-016-0155-7

**Published:** 2016-06-14

**Authors:** Jin-young Min, Kyoung-bok Min

**Affiliations:** Institute of Health and Environment, Seoul National University, Seoul, Republic of Korea; Department of Preventive Medicine, College of Medicine, Seoul National University, 103 Daehak-ro, Jongno-gu, Seoul, 110-799 Republic of Korea

**Keywords:** Cadmium, Alzheimer disease, Population health, Environmental, NHANES

## Abstract

**Background:**

Cadmium, a ubiquitous environmental pollutant, exhibits potential neurotoxic risk. Although compelling evidence suggests cadmium accumulation has a role in the formation of amyloid-β plaques and neurofibrillary tangles, which are the hallmarks of Alzheimer's disease (AD) pathogenesis, the supporting evidence in humans is limited and conflicting. In this study, we investigated the association between blood cadmium levels and AD mortality among older adults by analyzing the prospective data from the 1999–2004 Third National Health and Nutrition Examination Survey (NHANES) and the Linked Mortality File.

**Methods:**

The data were obtained from the 1999–2004 NHANES and the NHANES (1999–2004) Linked Mortality File. A total of 4,064 participants aged ≥60 years old with available blood cadmium data and no other missing information on their questionnaires at baseline were included in this study. AD was denoted as G30 based on the ICD-10 underlying causes of death.

**Results:**

Of the 4,064 participants, 51 subjects died as a result of AD. Compared with participants with low blood cadmium levels (≤0.3 μg/L), those with high cadmium levels (>0.6 μg/L) exhibited a 3.83-fold (hazard ratio = 3.83; 95 % CI = 1.39–10.59) increased risk of AD mortality. In the Kaplan–Meier survival curves for cumulative AD mortality, higher levels of blood cadmium showed marginally significant association with increased mortality at baseline and in patients over 60 years of age (*p* = 0.0684).

**Conclusions:**

We observed a significant association between blood cadmium levels and AD mortality among older adults in the US. Our findings suggest that environmental exposure to cadmium may be a risk factor for AD.

## Background

Alzheimer’s disease (AD) is a progressive neurodegenerative disease that is characterized by the deposition of amyloid-β (Aβ) plaques and neurofibrillary tangles in the brain [1, 2]. The cause of AD is multifactorial and involves genetic predisposition, advanced age, low education, and health behaviors (i.e., smoking and physical inactivity) [3]. There is strong consensus that environmental pollutants are implicated in the pathogenesis of AD, and that they play a role in the formation of amyloid plaques and neuronal damage [4].

Cadmium is a ubiquitous environmental pollutant that is released by various natural and anthropogenic activities (i.e., mining, fossil fuel combustion, and the production of batteries and pigments) in to the atmosphere [5]. Cadmium has received much attention because of adverse health effects on the kidneys and the skeletal system and its carcinogenic properties [6]. Compelling evidence suggests that cadmium may also confer a neurotoxic risk [7, 8]. Experimental studies have proposed that cadmium can induce cell death and apoptotic morphological changes in cerebral cortical neurons [9–11]. Human exposure to cadmium is associated with slowing neurobehavioral performance and lower cognitive scores for workers in an industry and also in the general population [12–14]. Some authors reported increased body-burdens of cadmium among AD patients relative to healthy individuals, [15–17], although reports are conflicting [15].

Given the neurological toxicity of cadmium and its possible association with AD, we wanted to explore whether environmental exposure to cadmium is a risk factor for AD. In this study, we investigated the association between blood cadmium levels and AD mortality among older adults by analyzing the prospective data from the 1999–2004 Third National Health and Nutrition Examination Survey and the Linked Mortality File.

## Methods

### Study population

We used data from the 1999–2004 NHANES and the NHANES (1999–2004) Linked Mortality Public File from the United States [18]. The latter was a follow-up study of mortality that matched records from NHANES with data in the National Death Index as of Dec. 31, 2011 [19]. The date and cause of death in the National Death Index were derived from death certificates. The NHANES Linked Mortality files are available in two-year increments (e.g., NHANES 1999–2000, NHANES 2001–2002, and NHANES 2003–2004).

From the 1999–2004 NHANES data and the linked morality file, we initially included 5,603 participants aged ≥60 years at the time of the initial survey. We excluded 868 older adults for whom blood cadmium levels or causes of death recode were unavailable; we also excluded 671 participants who had missing data for other variables. The cohort analysis presented in this study was based on 4064 older adults in the 1999–2004 NHANES. This study's protocol was approved by the institutional review board of Seoul National University Hospital (IRB No. E-1604-074-754).

### Variable of interest

For the measurement of blood cadmium, whole blood specimens were processed, stored, and shipped to the Division of Environmental Health Laboratory Sciences, National Center for Environmental Health, Centers for Disease Control and Prevention for analysis. Blood cadmium levels were measured by a PerkinElmer Model SIMAA 6000 (PerkinElmer, Norwalk, CT) simultaneous multi-element atomic absorption spectrometer with Zeeman background correction. The analytical laboratory followed extensive quality control procedures. Whole-blood materials from the National Institute of Standards and Technology Standard Reference Materials were used for external calibration.

International Classification of Diseases 10th Revision (ICD-10) codes were used for all causes of deaths, and the code for AD was G30 based on the ICD-10 underlying causes of death.

Baseline covariates were obtained from the interview data of the 1999–2004 NHANES and included age (60–69, 70–79, and 80–89 yr), sex, ethnic background (white, black, Hispanic or other), education (less than high school, high school graduate, college or more), family income (less than $20,000 or $20,000 or more), smoking status (current, former, or never), serum cotinine (≤0.022, 0.023-0.037, 0.038-0.297, or ≥0.30 ng/mL), and rice eating in the past 30 days (yes or no). Data regarding disease history were also collected. Hypertension was defined as systolic blood pressure ≥140 mmHg and diastolic blood pressure ≥ 90 mmHg, the use of antihypertensive drugs or previous physician-diagnosed hypertension. Diabetes was defined as a fasting plasma glucose level ≥6.99 mmol/L, a nonfasting plasma glucose level ≥ 11.1 mmol/L, current insulin use or a prior physician diagnosis of diabetes. Body mass index (BMI) was calculated by dividing measured weight in kilograms by measured height in meters squared and was categorized into four groups- underweight (<18.5 kg/m^2^), normal weight (18.5–24.9 kg/m^2^), overweight (25–29.9 kg/m^2^), and obese (≥30.0 kg/m^2^).

### Statistical analysis

To account for the complex sampling design, weighted estimates of the population parameters were computed using the NHANES Analytic and Reporting Guidelines. All the analyses were performed using the PROC SURVEY procedures in SAS 9.3 (SAS Institute, Cary, NC, USA).

The blood cadmium levels were log transformed to improve normality, and the geometric mean was calculated. Smoking-adjusted means (or least square means) were predicted values from a multiple regression equation, by treating never smokers as the reference group using the “LS means” option of the “PROC SURVEYREG” procedure. Significant differences in the demographic and clinical variables were evaluated using the chi-square test and tests for trends to compare variables across ordered categories of blood cadmium levels. We divided the study population by blood cadmium quartiles (≤0.3, 0.3–0.4, 0.4–0.6, and >0.6 μg/L). A Cox proportional hazards regression using “PHREG” procedure was conducted to evaluate the association of AD–related mortality with blood cadmium quartiles. We calculated the hazard ratio (HR) for the risk of AD mortality with respect to blood cadmium quartiles by comparing the first quartile, the 95 % confidence intervals (CIs), and the p-values for the trends. In the hazard model, age and age square were treated as continuous variables, and the analyses were adjusted for potential confounders: Model 1 was adjusted for age, age square, sex, ethnicity, education, family income, smoking, and serum cotinine, and Model 2 was further adjusted for rice eating, BMI, and history of diabetes and hypertension. We also generated the Kaplan-Meyer curves using the ‘DIRADJ’ option to obtain the direct adjusted survival curve by averaging the predicted survival functions for baseline covariates.

## Results

Table 1 presents the mean blood cadmium level among older adults by demographic characteristics. For the unadjusted mean distribution, older adults with higher blood cadmium levels were more likely to be 60–69 years old, females, and other ethnicity, to have low income, less education, and high serum cotinine levels, to be underweight, and to exhibit no history of diabetes and hypertension, and to be current smokers. The smoking-adjusted mean cadmium levels showed similar patterns, except for higher cadmium levels among subjects at 80–89 years old and with history of hypertension.

**Table 1:** Geometric mean (SE) blood cadmium levels of the participants (*n* = 4064) at baseline with results grouped by demographic characteristics

	No.	Unadjusted mean (SE)	*p*-value	Smoking-adjusted^a^ mean (SE)	*p*-value
Age, y					
60–69	1852	0.48 (0.01)	0.002	0.45 (0.01)	<.0001
70–79	1354	0.49 (0.01)		0.51 (0.01)	
80–89	858	0.52 (0.01)		0.58 (0.01)	
Gender					
Male	2024	0.48 (0.01)	0.002	0.44 (0.01)	<.0001
Female	2040	0.50 (0.01)		0.54 (0.01)	
Ethnicity					
White	2388	0.49 (0.01)	0.017	0.49 (0.01)	0.001
Black	603	0.47 (0.01)		0.46 (0.01)	
Hispanic	972	0.48 (0.01)		0.48 (0.02)	
Other	101	0.62 (0.04)		0.63 (0.04)	
Education					
Less than high school	1678	0.52 (0.01)	<.0001	0.54 (0.01)	<.0001
High school	956	0.48 (0.01)		0.50 (0.01)	
College or higher	1430	0.46 (0.01)		0.46 (0.01)	
Family income					
Less than $20,000	1689	0.54 (0.01)	<.0001	0.54 (0.01)	<.0001
$20,000 or more	2375	0.46 (0.01)		0.47 (0.01)	
Serum cotinine, ng/mL					
Q1 (≤0.022)	1037	0.41 (0.01)	<.0001	0.46 (0.01)	<.0001
Q2 (0.023–0.037)	1013	0.45 (0.01)		0.50 (0.01)	
Q3 (0.038–0.297)	1028	0.42 (0.01)		0.48 (0.01)	
Q4 (≥0.30)	986	0.75 (0.02)		0.54 (0.02)	
Rice eating					
Yes	864	0.49 (0.01)	0.317	0.50 (0.01)	0.449
No	3200	0.48 (0.02)		0.49 (0.01)	
Body mass index, kg/m^2^					
< 18.5	51	0.67 (0.05)	<.0001	0.54 (0.04)	<.0001
18.5–24.9	1106	0.56 (0.02)		0.54 (0.01)	
25.0–29.9	1646	0.47 (0.01)		0.48 (0.01)	
≥ 30	1261	0.45 (0.01)		0.47 (0.01)	
Diabetes					
Yes	876	0.45 (0.01)	0.003	0.45 (0.01)	0.003
No	3188	0.50 (0.01)		0.50 (0.01)	
Hypertension					
Yes	2760	0.49 (0.01)	0.568	0.51 (0.01)	0.003
No	1304	0.50 (0.01)		0.47 (0.01)	
Cigarette smoking					
Current smoker	494	1.14 (0.03)	<.0001		
Former smoker	1676	0.49 (0.01)			
Never smoker	1894	0.39 (0.00)			

^a^Smoking-adjusted mean levels were calculated by adjusting cigarette smoking status (current, former, and never smoker), in which never smokers were treated as the reference group

**Fig. 1:**
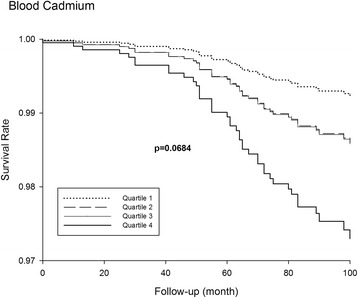
Kaplan–Meier survival curves for cumulative AD mortality according to blood cadmium quartile at baseline. Cadmium quartiles: Quartile 1: ≤ 0.3 μg/L; Quartile 2: 0.3–0.4 μg/L; Quartile 3: 0.4–0.6 μg/L; Quartile 4: > 0.6 μg/L. Quartiles 2 and 3 are almost indistinguishable

Table 2 presents the HRs for AD–related mortality by blood cadmium quartiles at baseline. After multivariable adjustment, older adults in the highest cadmium quartile exhibited an adjusted HR for AD mortality of 3.76 (95 % CI = 1.43–9.93) in Model 1 and 3.38 (95 % CI = 1.38–10.59) in Model 2, compared to those in the lowest cadmium quartile. In the Kaplan–Meier survival curves for cumulative AD mortality (Fig. 1), higher levels of blood cadmium showed marginally significant association with increased mortality at baseline and in adults older than 60 years of age (*p* = 0.0684).

**Table 2:** Hazard ratio (HR) for AD mortality by blood cadmium level at baseline

	Event no. (%)	Crude HR (95 % CIs)		Adjusted HR (95 % CIs)^a^			
Blood cadmium level				Model 1		Model 2	
Cadmium quartile, μg/L							
Quartile 1 (≤0.3)	7 (0.7)	1.00	(ref)	1.00	(ref)	1.00	(ref)
Quartile 2 (0.3–0.4)	11 (1.4)	1.80	(0.55–5.92)	1.83	(0.57–5.86)	1.81	(0.54–6.07)
Quartile 3 (0.4–0.6)	15 (1.3)	1.99	(0.83–4.75)	1.86	(0.74–4.70)	1.88	(0.73–4.85)
Quartile 4 (>0.6)	18 (1.6)	2.66	(1.06–6.63)	3.76	(1.43–9.93)	3.83	(1.38–10.59)
*p* value for trend			0.228		0.068		0.068

Note: CIs = confidence intervals, HR = hazard ratio, ref = reference

^a^Model 1: Adjusted for age, age square, gender, ethnicity, education, family income, smoking, and serum cotinine level

Model 2: Further adjusted for rice eating, body mass index, diabetes, and hypertension

## Discussion

We found that blood cadmium levels were significantly associated with AD-related mortality among older adults. Compared with participants who exhibited low blood cadmium levels (≤0.3 μg/L), those with high cadmium levels (>0.6 μg/L) exhibited a 3.83-fold increased risk of AD mortality, and significant dose–response relationships were observed for the risk of AD mortality.

AD is the most common type of dementia and becomes a major public health issue as the population ages. The World Alzheimer Report 2014 revealed that the number of people with dementia was estimated at 44 million, posing a substantial portion of global burden of disease [20]. Although AD pathogenesis is multifactorial and complex, studies suggest that toxic metals may represent a possible cause of AD [21–23]. There is growing evidence that cadmium plays a role in the accumulation of Aβ plaques and tau protein [24–26], as the main pathological components of AD [1, 2]. Syme and Viles (2006) found that Cd(II) caused the 1H NMR chemical shift of the Val12 γCH3 protons in Aβ (1–16) that is similar to that observed for Zn(II), which is known to be an important inducer of amyloid production [25, 27]. In a mouse model, relative to the control group, the cadmium-treated group exhibited lowered learning ability and reduced expression of α-secretase, soluble APPα and the neutral endopeptidase protein in the hippocampus and cerebral cortex, thereby contributing to the increases in the Aβ1-42 levels [28]. A recent study revealed the interaction between Cd(II) and the Aβ1-42 peptide; Cd(II) decreased Aβ1-42 peptide channel activity and turnover until the channel activity is completely abrogated [29]. Jiang et al. (2007) added evidence of the involvement of Cd(II) in the tau tangle formation through the acceleration or induction of the aggregation of Alzheimer's tau peptide R3 [24].

Few human studies have supported the possible link of cadmium with AD pathogenesis. Compared with normal subjects, elevated cadmium levels in the liver, plasma, and brain tissue [15–17] have been detected among AD patients. Our study, which demonstrated that the low blood cadmium levels currently observed in the US general population are associated with an increased risk for AD mortality, is consistent with the limited evidence. Admittedly, the reason for the observed association remains uncertain, but the potential relevance of cadmium to amyloid and tau filament formation may help explain the increased risk for developing AD.

The findings from the present study should be interpreted with caution. The most important limitation was that only a single blood cadmium measurement per patient was available. After cadmium exposure, it binds to metallothionein and accumulates in the human body, particularly in the kidney [30, 31]. Biomonitoring of cadmium exposure for environmentally exposed populations is usually conducted by detecting cadmium levels in the urine and blood. Urine cadmium level is a reflection of body burden (mainly kidney) with a half-life of 11.6 years [31]. In contrast, blood cadmium, a marker of ongoing exposure, reflects recent uptake. Its half-lives ranged from three to four months in the fast compartment and from 7.4 to 16.0 years in the slow component [32]. Blood cadmium levels used in the blood are dependent on daily exposure and primarily reflect recent exposure. Thus, we could not completely assess cadmium exposure. In addition, we relied only on death certificates obtained from the 1999–2004 NHANES Linked Mortality File and did not have information on the morbidity incident events. AD and other dementias were rarely reported as the primary cause of death [33, 34]. This is potentially because death certificates often list the immediate cause of death, rather than listing dementia as an underlying cause. In one series of AD cases, only 20.8 %–36 % of the total AD patients stated AD as the cause of death [33, 34]. Finally, we adjusted for several variables in the statistical model; because of the observational nature of this investigation, we cannot rule out the possibility of residual confounding effects by unmeasured confounders. Moreover, maximum 100 months follow up period is generally too short for the population without AD to compare the survival from AD deaths, which is reflected in the very high survival rate above 0.97 even in the highest category of the bold cadmium subgroups.

## Conclusion

This study showed a significant association between blood cadmium levels and AD mortality among older adults in the US. Although further studies are necessary to clarify the observed association, our findings support the potential relevance of cadmium to AD pathogenesis and suggest that the elevation of blood cadmium levels may present an important predictive marker of increased AD mortality risk. Thus, efforts to reduce cadmium-related disease risk from environmental exposure to cadmium should be continued.

## Abbreviations

AD, Alzheimer's disease; Aβ, amyloid-β; CIs, confidence intervals; ICD-10, International Classification of Diseases 10th Revision; HR, hazard ratio; NHANES, National Health and Nutrition Examination Survey
